# Echocardiographic imaging of tricuspid and pulmonary valve abnormalities in primary ovarian carcinoid tumor

**DOI:** 10.1186/1476-7120-8-37

**Published:** 2010-09-02

**Authors:** Constantina Aggeli, Ioannis Felekos, Christina Kazazaki, Dimitrios Giannopoulos, Athanasios Kartalis, Christos Pitsavos, Christodoulos Stefanadis

**Affiliations:** 1 1st Department of Cardiology, University of Athens Medical School, Hippokration Hospital, Athens, Greece

## Abstract

Carcinoid is a rare malignancy originating from enterochromaffin cells and is clinically characterized by flushing, diarrhea and bronchospasm, due to secretion of vasoactive substances. A dreaded complication is carcinoid heart disease, which mainly affects right cardiac chambers, resulting in thickened, immobile and retracted tricuspid and pulmonary valves. In the current report, a case of a 60-year old female presenting with symptoms of right heart failure is described. Transthoracic two-dimensional and real-time three-dimensional echocardiography findings, as well as biochemical markers, including pro-BNP and NT-pro-BNP, were consistent with carcinoid syndrome. The histological diagnosis of carcinoid was confirmed after surgical resection of an ovarian mass.

## Background

Primary cardiac tumors are rare (5%) while metastatic tumors of the heart, predominantly from carcinoma of lung and breast, malignant melanoma, and leukemias and lymphomas, constitute the majority of cardiac tumors

Cardiac carcinoid is an exceedingly rare cause of valvular disease and is characterized by plaque-like deposits of fibrous tissue on endocardial surface of valve cusps and leaflets usually on the right heart valves (90%).

A case, regarding an ovarian carcinoid mass with cardiac manifestations involving both tricuspid and pulmonary valves, is described.

## Case Presentation

A 60-year old Caucasian female, was referred to our cardiology department, due to early fatigue (NYHA II/III) and ankle edema, progressively deteriorating over the last year, also complaining of anorexia. She had a medical history of hypertension, which was treated with an angiotensin receptor blocker, with no other risk factors for coronary artery disease. The rest of her medical history was unrevealing.

On physical examination, the patient had 90 bpm and a respiratory rate of 12 breaths/min. Her temperature was 37°C and her blood pressure was 140/90 mmHg. Cardiac examination revealed a left parasternal holosystolic murmur, a third cardiac sound (S3) and a palpable right ventricular heave, whereas lung auscultation was unremarkable. The rest of the physical examination indicated signs of right heart failure, including palpable liver, elevated jugular pressure and pitting peripheral edema.

**Figure 1:**
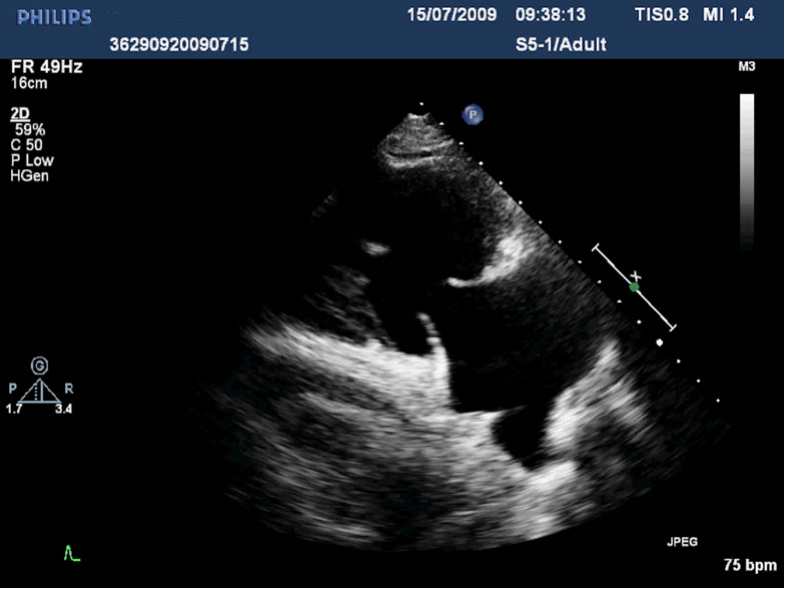
**Right ventricular inflow view in systole showing thickened, immobile and retracted anterior and septal leaflets of tricuspid valve**.

**Figure 2:**
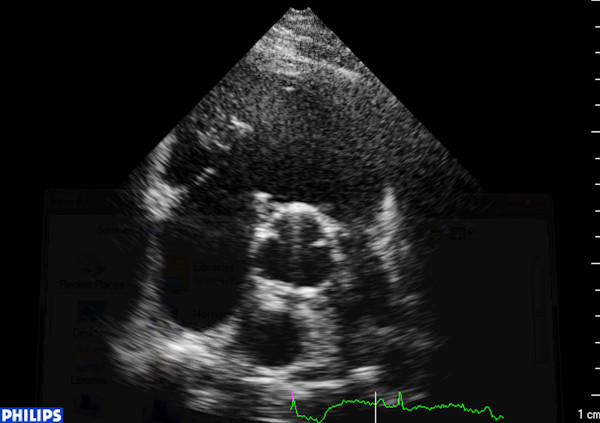
**Short axis view in diastole depicting a fixed and immobile pulmonary valve**.

**Figure 3:**
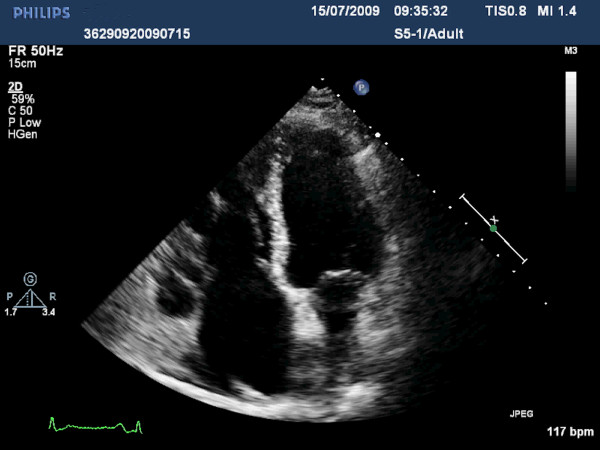
**Apical four-chamber view in systole displaying an opened and retracted tricuspid valve, while mitral valve is closed**.

**Figure 4:**
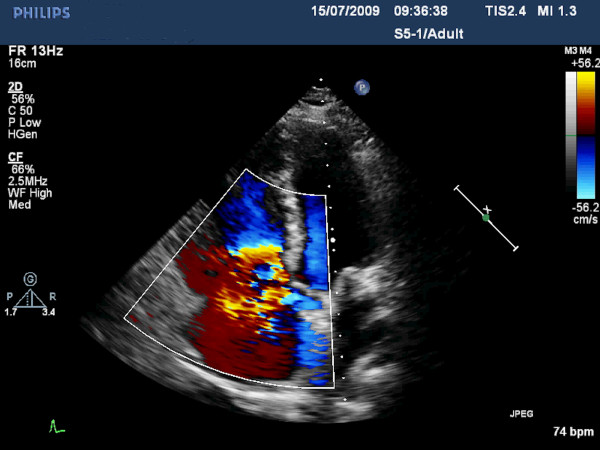
**Apical four-chamber view in diastole**. The color Doppler demonstrates severe tricuspid valve regurgitation.

**Figure 5:**
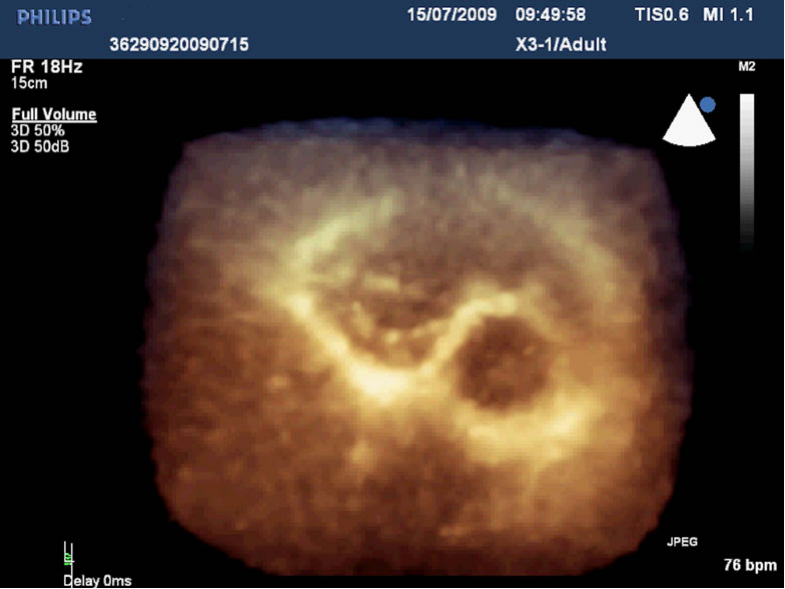
**Real-Time three-dimensional image showing the thickened and retracted tricuspid valve from the perspective of the right atrium**.

The electrocardiogram revealed incomplete right bundle branch block, while the initial biochemical control showed increased levels of natriuretic peptides Nt-proBNP (800 pg/ml) and BNP (387 pg/ml). According to two-dimensional and real-time three-dimensional transthoracic echocardiographic study, left cardiac chambers had normal size and function and the respective valves moved normally throughout the cardiac cycle. In contrast, enlarged and dilated right cardiac chambers with mildly impaired systolic function were recorded. The leaflets of tricuspid and pulmonary valve were thickened, immobile and retracted, leading in valve malcoaptation and consequent severe regurgitation, as showed by the color Doppler (Figures 1, 2, 3, 4) (See additional files 1, 2, 3, 4, 5). Moreover three-dimensional echo provided unique en-face views of the thickened tricuspid valve leaflets which were fixed in a semiopen position, causing a large area of noncoaptation (Figure 5) (See additional file 6). The clinical and echocardiographic findings aroused the suspicion of carcinoid heart disease. Levels of 24-hour urine 5-HIAA were increased (572 μmol/24 h), a finding consistent with carcinoid disease. Due to impaired right ventricular function, the surgical replacement of both tricuspid and pulmonary valves was not recommended.

**Figure 6:**
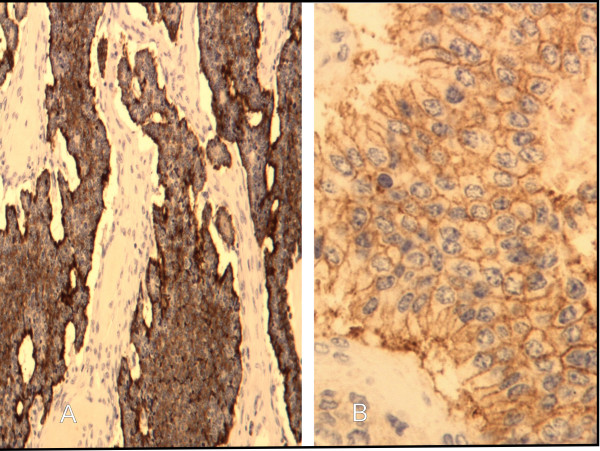
**Well differentiated neuroendocrine tumor**. (A) Positive immunohistochemical stain for Chromogranin. (B) Positive immunohistochemical membranal stain for CD56.

Moreover, the patient's abdominal CT revealed a mass in the ovary and she was operated. Primary ovarian carcinoid was histologically revealed (Figure 6).

## Discussion

Carcinoid tumors are uncommon malignancies that arise from enterochromaffin cells typically located in the gastrointestinal tract or lungs [1]. These tumors may secrete large amounts of vasoactive substances, including 5-hydroxytryptamine and prostaglandins, which in turn cause various clinical manifestations such as flushing, diarrhea, and bronchospasm. Primary ovarian carcinoid tumors are rare and unique, due to the fact that they release vasoactive substances directly into the inferior vena cava (right ovary) or renal vein (left ovary), entering the systemic circulation [2]. Cardiac lesions are found in 50-60% of patients with carcinoid syndrome, usually between 18-24 months after diagnosis is established [3]. Patients with carcinoid heart disease typically present with symptoms of right-sided heart failure (hepatomegaly, edema, ascites, fatigue, and low cardiac output), and when symptoms are advanced (NYHA class 3 or 4), outlook is poor [4,5].

Diagnosis can be made relying on biochemical markers and imaging techniques [6]. Increased levels of natriuretic peptides Nt-proBNP, BNP and 24-hour urine 5-HIAA are typical of this disease. Moreover, according to various studies, there seems to be a strong correlation between serotonin, 5-HIAA levels and the prevalence of carcinoid heart disease [7,8]. It is known that plaque-like fibrous endocardial thickening leads to cardiac valve involvement. Echocardiography is the most commonly used modality in this setting, revealing retraction and/or fixation of the valves, findings that are pathognomonic for the specific entity. Although the role of newer imaging modalities has not yet been established, three-dimensional echocardiography may play an incremental role for valvular lesion and right ventricular evaluation in patients with carcinoid heart disease [9,10]. An alternative modality would be cardiac magnetic resonance providing accurate anatomic and functional information, not only for cardiac chambers but also for heart valves [11]. Moreover, magnetic resonance imaging has the advantage over CT that the tricuspid valve motion and valvular dysfunction can be precisely assessed and quantified using cine MRI in combination with velocity-encoded cine MRI [12,13]. To this point, an integrated approach utilizing multimodality imaging, in order to assess the severity of disease, is perhaps the most suitable management strategy [13].

Treatment constitutes mainly of somatostatin analogs, which inhibit the release of various biogenic amines and peptides, including serotonin, and as a result there is a marked alleviation of symptoms [6]. Cardiac surgery should be considered early for patients with symptomatic carcinoid valve disease and controlled carcinoid symptoms [14], as disease progression may have an unfavourable impact on surgery and survival outcomes.

## Conclusion

This is a rare case of cardiac carcinoid, because of the unusual primary tumor origin in addition to the dual valve involvement. Primary ovarian carcinoid accounts only for 0.1% of all ovarian malignancies and less than 5% of total carcinoid tumors [15]. On the other hand, carcinoid usually affects the right cardiac chambers leading in thickening and retraction of the respective valves, with the tricuspid valve being the most commonly affected structure. Therefore, the current report, where both tricuspid and pulmonary valve are involved, consists a minority of carcinoid valve disease cases. Echocardiography is the modality of choice for the assessment of this entity, with real-time three dimensional echo providing unique en face views of the complex anatomy of the cardiac valves and the valvular apparatus. In the present clinical case, the combination of echocardiographic imaging and circulating biomarkers provided useful insights, leading accurately and undoubtedly to the diagnosis, which was subsequently verified by the histological study. In our patient, although tricuspid and pulmonary valves had severe regurgitation, the surgical replacement was not feasible, due to impaired right ventricular systolic function.

## Competing interests

The authors declare that they have no competing interests.

## Authors' contributions

CA conceived the case report, performed bedside echocardiographic examinations, IF reviewed literature and wrote the manuscript. CK and AK have been involved in drafting the manuscript. DG has provided the histological analysis. CP contributed critical revision of the manuscript. CS has supervised and commented the manuscript. All authors read and approved of the final manuscript.

## Consent

Written informed consent was obtained from the patient's legal representative for publication of this case report and any accompanying images. A copy of the written consent is available for review by the Editor-in-Chief of this journal.

## Supplementary Material

Additional file 1 **Video 1**. Right ventricular inflow tract showing thickened, immobile and retracted anterior and septal leaflets of the tricuspid valve. Click here for file

Additional file 2 **Video 2**. Short axis view depicting fixed and immobile tricuspid and pulmonary valves. Click here for file

Additional File 3 **Video 3**. Apical four-chamber view displaying an opened and retracted tricuspid valve with normal mitral valve function, enlarged right ventricle and dilated right atrium. Click here for file

Additional file 4 **Video 4**. Apical four-chamber view. The color Doppler demonstrates severe tricuspid valve regurgitation Click here for file

Additional file 5 **Video 5**. Short axis view depicting severe pulmonary valve regurgitation. Click here for file

Additional file 6 **Video 6**. Real-Time three-dimensional image showing the thickened and retracted tricuspid valve from the perspective of the right atrium, while mitral valve moves normally throughout the cardiac cycle. Click here for file
